# Betulin‐Containing Gel in the Management of a Patient With Recurrent Superinfected Wounds in Chronic Prurigo: A Case Report

**DOI:** 10.1111/iwj.70909

**Published:** 2026-04-14

**Authors:** Jan Nicolai Wagner, Franziska Zirkenbach, Matthias Augustin

**Affiliations:** ^1^ Institute for Health Services Research in Nursing and Dermatology (IVDP), University Medical Center Hamburg‐Eppendorf (UKE) Hamburg Germany

**Keywords:** birch bark extract, chronic prurigo, chronic wounds, superinfection, topical therapy, wound healing, wound infection

## Abstract

Chronic prurigo is frequently complicated by chronic wounds and secondary bacterial infections, representing a major therapeutic challenge. A betulin‐containing gel, approved for wound treatment in epidermolysis bullosa, has demonstrated wound‐healing and antimicrobial properties beyond its licensed indication. To describe the clinical course of a chronic, recurrently superinfected wound in chronic prurigo treated with a betulin‐containing topical gel. A 53‐year‐old male with chronic prurigo under systemic therapy with the Interleukin‐4 and ‐13 antibody Dupilumab, presented with a persistent ulcerative lesion at the mandibular angle, repeatedly colonized by pathogenic bacteria despite prior systemic and topical antimicrobial therapy. Betulin‐containing gel was applied twice daily under occlusion for 3 weeks. Clinical outcomes and microbiological findings were assessed. After 3 weeks of treatment, marked clinical improvement was observed, including reduced erythema and crusting, resolution of pain and itch, and complete epithelial stabilization. Follow‐up wound swabs were negative for pathogenic bacteria. This case suggests that betulin‐containing gel may represent a useful adjunctive treatment option for complex, superinfected chronic wounds in chronic prurigo. Further controlled studies are required to clarify its role in multimodal wound management.

## Key Points

- Chronic prurigo is frequently complicated by persistent, superinfected wounds that remain refractory despite adequate systemic control of inflammation and repeated antimicrobial therapy.
- In this case, a chronic ulcerative lesion with recurrent bacterial colonization showed rapid and sustained clinical improvement following 3 weeks of treatment with a betulin‐containing gel applied under occlusion.
- Treatment was associated with complete epithelial stabilization, resolution of pain and pruritus, and clearance of pathogenic bacteria without the use of concomitant antibiotics.
- The observed effects may be attributable to combined wound‐healing, barrier‐stabilizing, anti‐inflammatory, and indirect antimicrobial properties of betulin.
- Betulin‐containing gel may represent a valuable non‐antibiotic adjunct in the multimodal management of complex wounds in chronic prurigo, potentially reducing reliance on repeated antibiotic therapies.
- Controlled studies are needed to confirm efficacy, clarify mechanisms of action, and define its role in chronic inflammatory dermatoses with secondary infection.

## 1 Introduction

Chronic prurigo (CPG) is a chronic inflammatory skin disease characterized by persistent itching and the presence of multiple puriginous lesions. According to the European Prurigo Project, the diagnosis of CPG is based on chronic itch lasting longer than 6 weeks, a history and/or signs of repeated scratching, and the development of characteristic puriginous lesions. These lesions are frequently excoriated and may become secondarily infected as a result of ongoing mechanical trauma. These complications often result in delayed wound healing, repeated use of antibiotics and a lower quality of life [1, 2].

In 2022, the European Medicines Agency (EMA) approved a betulin‐containing gel, a refined dry extract from birch bark, for treating wounds in patients with epidermolysis bullosa [3]. It improves wound healing by promoting keratinocyte differentiation, stimulating re‐epithelialization and modifying inflammatory and oxidative stress pathways [4, 5, 6]. Experimental and clinical data suggest that these mechanisms may also be relevant in other wound entities, including burn wounds, postsurgical wounds, diabetic ulcers and virus‐associated skin lesions [7, 8, 9, 10, 11].

However, there is limited evidence for using betulin‐containing gel in chronic inflammatory skin conditions complicated by repeated infections. In this report, we present a case of a long‐lasting, recurrently infected chronic wound in a patient with chronic prurigo that was successfully treated with topical betulin‐containing gel.

## 2 Material and Methods

### 2.1 Study Design and Patient

This report describes a single‐patient observational case study conducted in routine clinical practice. A 53‐year‐old male with a long‐standing history of CPG was followed at a dermatology university outpatient clinic.

### 2.2 Clinical Background

The patient had been treated with the interleukin‐4 and interleukin‐13 inhibitor dupilumab since 2023, resulting in near‐complete remission of generalized pruritus and pruriginous nodules (worst itch numeric rating scale (NRS) reduced from 10/10 to 2/10). However, a solitary ulcerative lesion measuring approximately 2 × 2 cm persisted at the left mandibular angle. At baseline, the persistent lesion was associated with localized pruritus (worst itch NRS 8/10) and mild pain (skin pain NRS 2/10). The patient reported frequent unconscious scratching and manipulation of the lesion, particularly during stress and at night. Relevant comorbidities included bronchial asthma, currently not requiring pharmacological treatment.

### 2.3 Previous Treatments

The lesion had been present intermittently for several months and was repeatedly colonized by *Staphylococcus aureus* (fusidic acid‐resistant) and subsequently *Klebsiella aerogenes* . Prior treatment included topical gentamicin ointment and oral cefadroxil (1 g twice daily for 7 days), with only transient clinical improvement.

### 2.4 Intervention

Given the chronicity and repeated superinfection, treatment with a betulin‐containing gel was initiated. The gel was applied topically twice daily under occlusion for a total duration of 3 weeks. No additional topical antimicrobial agents were used during this period.

### 2.5 Outcome Assessment

Clinical assessment focused on erythema, crusting, pain and pruritus. Microbiological wound swabs were obtained before and after treatment to assess bacterial colonization. Swabs were taken as routine superficial wound swabs according to local clinical practice.

## 3 Ethics

According to local regulations, formal ethics committee approval was not required for this observational case report. Written informed consent for treatment and publication of clinical images was obtained from the patient.

## 4 Results

**FIGURE 1:**
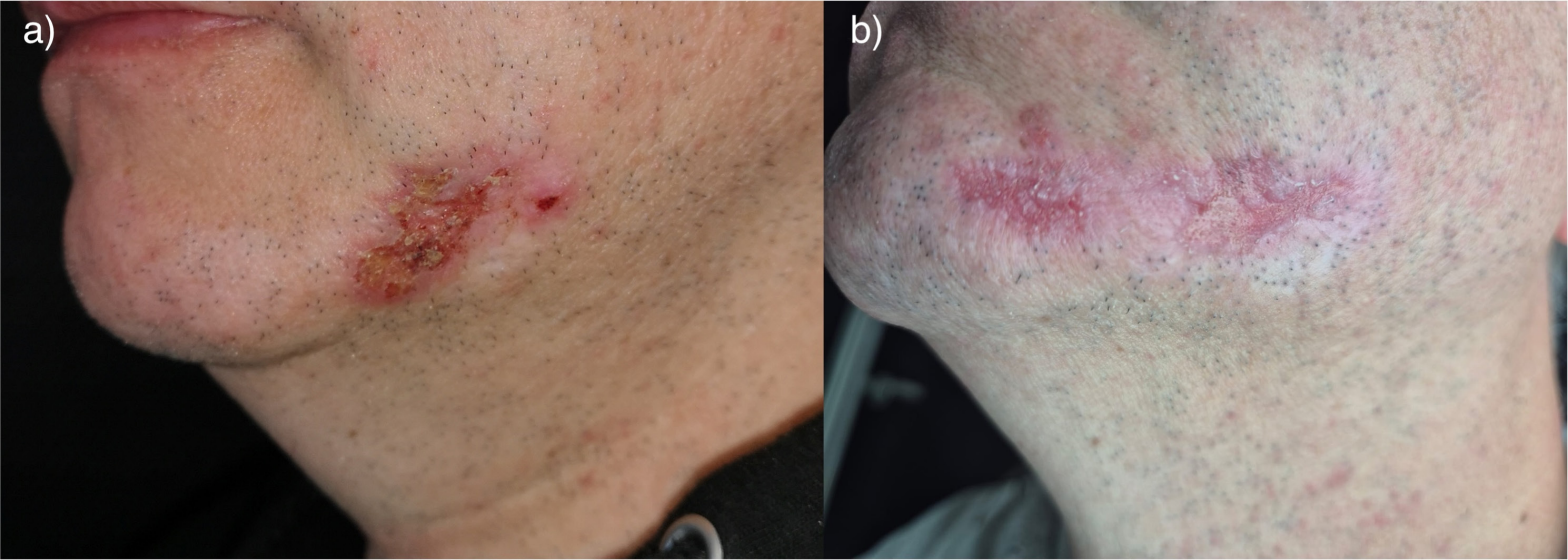
Clinical image before treatment (a). Marked regression of the lesion after 3 weeks of treatment (b).

At baseline, the lesion showed pronounced erythema, crusting, tenderness and pruritus. Microbiological swabs confirmed bacterial colonization. After 3 weeks of treatment with betulin‐containing gel, marked clinical improvement was observed. The lesion demonstrated substantial epithelial stabilization with reduced erythema and crusting. Pain and itch had fully resolved. Follow‐up microbiological swabs were negative for pathogenic bacteria (Figure 1a,b). No local systemic adverse effects were observed during the treatment period.

## 5 Discussion

This case illustrates the potential utility of betulin‐containing gel as an adjunctive treatment for complex, recurrently superinfected chronic wounds in patients with CPG. Despite effective systemic control of inflammation with dupilumab, the persistent wound remained refractory to standard topical and systemic antibiotic therapy. The facial localization is atypical for CPG, which predominantly affects the extensor surfaces of the extremities; however, persistent manipulation and scratching behaviour likely contributed to the maintenance of this solitary lesion.

Beyond its wound‐healing properties, betulin‐containing gel may exert anti‐inflammatory effects that are relevant in chronic inflammatory dermatoses. Experimental data suggest modulation of inflammatory pathways, reduction of oxidative stress and promotion of keratinocyte differentiation, which may contribute to stabilization of the epidermal barrier. In the context of CPG, these mechanisms may help to interrupt the scratch cycle by reducing local inflammation and facilitating re‐epithelization [4, 5, 6].

The cutaneous microbiome may also play a relevant role. Dysbiosis and colonization with pathogenic bacteria can exacerbate inflammation and pruritus. Recent data highlight alterations in the skin microbiome in prurigo nodularis, suggesting that microbial factors may contribute to disease persistence [12, 13]. In this context, the antimicrobial and barrier‐stabilizing properties of betulin may indirectly support restoration of microbial balance.

Importantly, conclusions regarding causality cannot be drawn from this single‐patient observation, and the lack of a control limits interpretation. Nevertheless, spontaneous resolutions appear unlikely given the long‐standing, recurrent nature of the lesion and its persistence despite prior standard antimicrobial therapies, with sustained epithelization and pathogen clearance occurring only after initiation of betulin‐containing gel. While contributory effects of occlusion cannot be fully excluded, the temporal association and chronic refractory course support a treatment‐related effect.

From a wound perspective, chronic prurigo‐associated wounds present a challenging entity due to ongoing mechanical irritation and secondary infection. Current wound infection guidelines emphasize the importance of restoring barrier function and minimizing unnecessary antibiotic exposure [14, 15]. In this context, non‐antibiotic topical agents with barrier‐stabilizing and wound‐healing properties may represent valuable adjuncts in multimodal wound management strategies.

## 6 Conclusion

Topical betulin‐containing gel may represent a promising adjunct in the multimodal management of complex, recurrently superinfected chronic wounds in CPG. Further exploration is warranted into the role of birch bark extract in promoting an environment less favourable to bacterial persistence and mechanical perpetuation of the itch‐scratch cycle, especially in conditions such as CPG, where manipulation and secondary infection frequently complicate wound healing [15].

## Funding

This work was supported by Open Access Publication Fund of UKE—Universitätsklinikum Hamburg‐Eppendorf.

## Consent

The patient has signed written formal consent to publication of medical history and clinical picture.

## Conflicts of Interest

Matthias Augustin: M.A. has served in an advisory role for Chiesi. Jan Nicolai Wagner and Franziska Zirkenbach declare no conflicts of interest.

## Data Availability

The data that support the findings of this study are available from the corresponding author upon reasonable request.
